# Sad Facial Expressions Increase Choice Blindness

**DOI:** 10.3389/fpsyg.2017.02300

**Published:** 2018-01-08

**Authors:** Yajie Wang, Song Zhao, Zhijie Zhang, Wenfeng Feng

**Affiliations:** ^1^Department of Psychology, School of Education, Soochow University, Jiangsu, China; ^2^Department of Psychology, Hebei Normal University, Shijiazhuang, China

**Keywords:** choice blindness, facial expressions, sad faces, happy faces, neutral faces

## Abstract

Previous studies have discovered a fascinating phenomenon known as choice blindness—individuals fail to detect mismatches between the face they choose and the face replaced by the experimenter. Although previous studies have reported a couple of factors that can modulate the magnitude of choice blindness, the potential effect of facial expression on choice blindness has not yet been explored. Using faces with sad and neutral expressions (Experiment 1) and faces with happy and neutral expressions (Experiment 2) in the classic choice blindness paradigm, the present study investigated the effects of facial expressions on choice blindness. The results showed that the detection rate was significantly lower on sad faces than neutral faces, whereas no significant difference was observed between happy faces and neutral faces. The exploratory analysis of verbal reports found that participants who reported less facial features for sad (as compared to neutral) expressions also tended to show a lower detection rate of sad (as compared to neutral) faces. These findings indicated that sad facial expressions increased choice blindness, which might have resulted from inhibition of further processing of the detailed facial features by the less attractive sad expressions (as compared to neutral expressions).

## Introduction

It is commonly believed that the outcome of a choice stands for individual preference. Based on this assumption, if the outcome of our choice is replaced with another outcome, then we should easily detect this mismatch because the replaced outcome would contradict our original preference. However, is this always the case? Previous studies discovered a fascinating phenomenon known as choice blindness (CB), wherein people fail to notice a radical change to the outcome of their choice (Johansson et al., 2005). In a typical choice blindness experiment, a pair of female faces was presented, and participants were asked to choose the more attractive face. Later, when participants were confronted with their choice, they were asked to report the reasons for choosing it verbally (non-manipulated trials). In certain trials, participants' choices were reversed, so that they were confronted with the opposite outcome of their intended choice (manipulated trials). Surprisingly, in the majority of the manipulated trials, participants not only failed to detect the mismatches between their preferences and the outcomes but also were prepared to describe the reasons why they chose the face that they never intended to choose (Johansson et al., 2005). Furthermore, using various linguistic markers (certainty, specificity, emotionality, complexity, etc.), comparisons of introspective reasons that were reported verbally showed no significant difference between manipulated and non-manipulated trials (Johansson et al., 2005, 2006).

Although the robust effect of CB was consistently observed in a series of later studies (Johansson et al., 2005, 2006; Sagana et al., 2013; Sauerland et al., 2016; Somerville and McGowan, 2016), several behavioral studies have reported a couple of factors that can modulate the extent of choice blindness. For example, there was a higher detection rate (i.e., lower magnitude of choice blindness) in the free time condition compared to the fixed viewing time (e.g., 5 s of deliberation time) condition (Johansson et al., 2005). Besides, choice blindness was significantly increased for faces with higher similarity (Sagana et al., 2013). Furthermore, there was a substantial reduction in the incidence of choice blindness when participants displayed a strong overall preference for the presented stimuli (Somerville and McGowan, 2016). It is noteworthy that previous choice blindness studies using faces as stimuli selected only faces with neutral facial expression as stimuli. However, human faces have great biological/social importance, especially facial expressions play an important role in decision-making and social interaction (Hansen and Hansen, 1988; Fox et al., 2000; Seymour and Dolan, 2008; Ebner et al., 2010), and previous studies, to our knowledge, have not yet investigated the potential effects of facial expressions on choice blindness. Hence, the aim of the present study was to investigate whether choice blindness could be influenced by facial expressions.

As mentioned above, the task in the choice blindness paradigm was to choose which face in each pair was more attractive; in other words, participants made their choices probably based on the evaluation of facial attractiveness. Previous studies have shown that individuals appear less physically attractive when their facial expression is sad when compared with being neutral or happy (Byrne and Clore, 1970; Mueser et al., 1984; Gelstein et al., 2011; Morrison et al., 2013), which is probably because a sad facial expression is more likely to be a distressing cue, resulting in participants reacting to the sad expression with avoidance-related behaviors (Seidel et al., 2010). It has also been found that more attractive faces (i.e., happy or neutral faces) are recognized more easily in a recognition task (Tsukiura and Cabeza, 2008; Marzi and Viggiano, 2010). This attractiveness effect on recognition was found to be related to enhanced reward signals processed by the orbitofrontal cortex, which contributed to encoding and retrieval processing stages (Tsukiura and Cabeza, 2008; Marzi and Viggiano, 2010).

According to the previous findings, we hypothesized that the processing of facial expressions might influence the magnitude of choice blindness by modulating facial attractiveness, and higher choice blindness would be observed for a negative expression than for positive and neutral expressions. To test these hypotheses explicitly, happy, neutral, and sad female faces were used as stimuli in the present study. Using the established CB paradigm and a similar card-trick methodology (manipulation) described by Johansson et al. (2005), participants were asked to choose the more attractive face in each pair under each of the two facial expression conditions (neutral and sad expressions for subjects who participated in Experiment 1, and neutral and happy expressions for those who participated in Experiment 2). In addition, the same detection classification that was used in Johansson et al.'s study (Johansson et al., 2014), including concurrent and retrospective detection, was introduced to the present study.

## Materials and methods

### Participants

A total of 120 students (61 females, mean age 22.3 years, *SD* = 3.6) participated in the study after giving written informed consent as approved by the ethical committee of the Soochow University. All participants were undergraduates. Both the experiments comprised 60 participants each. All participants were naive about the purpose of the study and reported normal or corrected-to-normal vision. Each of them received a small payment in return for their participation. All procedures for the current study were approved by the ethical committee of Soochow University.

### Stimuli

The experiments were implemented by “E-prime” software (version 2.0) on a portable computer (Lenovo Z380) with a 13.3-inch monitor (refresh rate 60 Hz, resolution 1,366 × 768). Forty-five pairs of grayscale photographs of female faces from the Chinese Affective Picture System (CAPS, Gong et al., 2011) were chosen as stimuli, including 15 pairs of sad faces, 15 pairs of neutral faces, and 15 pairs of happy faces, respectively. Each photograph showed a face in roughly frontal view with the size of 370 × 556 pixels.

In Experiment 1, 15 pairs of sad faces and 15 pairs of neutral faces were presented to the participants pair by pair, separately for sad faces and neutral faces. To avoid our results being attributed to differences in the similarity of paired faces between sad and neutral expressions, an attempt was made to keep the physical similarity constant at an intermediate level. The 15 pairs of sad faces and 15 pairs of neutral faces used in Experiment 1 were rated by 35 adults for similarity on a scale from 1 to 9 (1 = not at all similar; 9 = very similar) in a pilot study. The pairs of sad faces had a mean similarity of 4.40 (*SD* = 0.99; 95% CI = [4.06, 4.72]) and the pairs of neutral faces had a mean similarity of 4.62 (*SD* = 0.93; 95% CI = [4.30, 4.94]). There was no significant difference in similarity between sad and neutral faces [*t*_(34)_ = 1.33, *p* = 0.194, *d* = 0.22]. Three pairs of faces in each expression were chosen as target pairs (manipulated trials), in which participants received the opposite outcome of what they intended. The target pairs were always presented at the same position (the 7th, 10th, and 14th pairs) in the sequence (Johansson et al., 2005). It should be noted that no significant difference was observed on the similarity between target pairs of sad faces and neutral faces [*t*_(34)_ = 1.56, *p* = 0.129, *d* = 0.26].

In Experiment 2, 15 pairs of happy faces and the same 15 pairs of neutral faces as used in Experiment 1 were presented pair by pair, separately for happy faces and neutral faces. Another 35 raters used a nine-point scale to rate the similarity of the 15 pairs of happy faces and the 15 pairs of neutral faces. The happy face pairs had a mean similarity of 4.49 (*SD* = 1.15; 95% CI = [4.09, 4.89]), and the neutral face pairs had a mean similarity of 4.77 (*SD* = 0.78; 95% CI = [4.50, 5.04]). No significant difference was observed on the similarity between happy and neutral faces [*t*_(34)_ = 1.30, *p* = 0.203, *d* = 0.22]. Similar to Experiment 1, three pairs of faces in each expression were manipulated, and there was no significant difference on the similarity between these target pairs of happy faces and neutral faces [*t*_(34)_ = 1.26, *p* = 0.216, *d* = 0.21].

### Procedure

In Experiment 1, there were two experimental phases. In the first phase, 15 trials of sad faces were presented in one block, and 15 trials of neutral faces were presented in another block. Each block consisted of three manipulated trials (M trials) and 12 non-manipulated trials (NM trials). The manipulated trials were presented at the position of the 7th, 10th, and 14th in the trial sequence for both the blocks.

In each trial, participants were asked to decide the more attractive face in each pair by pressing “F” or “J” on the keyboard. Button F represented “choosing the left face,” and button J represented “choosing the right.” Each pair was presented on the screen for 4 s, and then it disappeared. Participants were instructed to choose the face that they found more attractive as soon as the photographs disappeared. The 4-s presentation of each face pair was chosen because previous studies had shown that this duration was sufficient for participants to form a preference for face pairs (Johansson et al., 2005; Willis and Todorov, 2010). Following the response, the exact outcome of the choice in non-manipulated trials was presented, and the subjects were asked to describe the reasons (any reason was fine) in oral form for their choices in Chinese. The reasons that participants delivered in each trial were recorded by the experimenter as “verbal reports.” For manipulated trials, however, participants would receive the *opposite* outcome of their choice after their response. If participants immediately detected that their choice outcome was reversed, this detection of M trial was recorded and classified as a “concurrent detection,” and they were asked to describe the reasons for this detection (verbal reports). If participants did not detect the ongoing M trial, they were also asked to deliver verbal reports for their choices (the same as in non-manipulated trials) when seeing the manipulated outcome.

As described above, the 15 trials of sad faces were presented to participants in one block, and the 15 trials of neutral faces were presented in another block. The reason for assigning sad faces and neutral faces into two separate blocks was that randomizing sad face pairs and neutral face pairs in one block might weaken the perceived difference in facial expression between sad and neutral faces and thus decrease the effect of facial expression (e.g., if sad faces were presented in the previous trial and neutral faces were presented in the current trial, then chances were that the perceived sad expression in the previous trial might lead to a tendency toward perceiving current neutral faces as sad expressions to some degree). To balance the possible effect of the block order (i.e., successfully detecting the manipulated trial(s) in the former block might lead to higher detection rate in the latter block, as participants might be aware of the manipulated trials in the former block and then pay more attention to differences between the two faces in the latter block), the order of the two blocks were counterbalanced across participants. The 15 pairs of sad faces were presented in the first block to half of the participants, and the 15 pairs of neutral faces were presented in the first block to the other half of the participants. In addition, an interference task was also introduced between blocks to minimize the possible effect of block order, in which the participants were asked to do a simple numerical addition and subtraction task for 5 min.

After the completion of the first phase (i.e., two blocks), the second experimental phase was an interview in which the participants were asked a series of questions (**Appendix**) about the experiment in the first phase. These questions aimed at determining whether participants had realized the manipulation but did not report it. If the participants declared that there was something strange with the face pairs, they were then asked to look through all face pairs again and to pick out the face pair(s) they thought had been manipulated. If they successfully picked out a manipulated face pair that had not been reported concurrently in the first phase, this detection would be recorded and classified as a “retrospective detection.”

The procedure of Experiment 2 was identical to Experiment 1 with the following exception: 15 trials of neutral face pairs (the same 15 pairs of neutral faces that were used in Experiment 1) and 15 trials of happy face pairs were presented to another 60 participants.

### Design

The dependent variable in the present study was the percentage of detected manipulated trials (see the Data analysis section for details). In Experiment 1, the independent variables were block order (between-subject factor: sad-face first vs. neutral-face first) and facial expression (within-subject factor: sad faces vs. neutral faces). In Experiment 2, block order (between-subject factor: happy-face first vs. neutral-face first) and facial expression (within-subject factor: happy faces vs. neutral faces) served as the independent variables.

### Data analysis

To investigate the effect of facial expressions on choice blindness for each participant, the detection rate of manipulated trials was separately calculated for neutral faces and sad faces in Experiment 1 and for neutral faces and happy faces in Experiment 2. The detection rate was calculated as the percentage of detected M trials, including concurrent detection (participants detected immediately after the originally chosen image was replaced) and retrospective detection (participants described they had experienced something strange and picked out the manipulation during the interview phase). The detection rate was subject to a two-way ANOVA with the factors of facial expression (neutral faces vs. sad/happy faces) and block order (neutral faces first vs. expressional faces first), separately, for Experiment 1 and Experiment 2. The potential effect of the block order was included in the statistical analysis to examine whether the facial expression effect (if there were any) would be accounted for by the block order [i.e., the effectiveness of the between-block interference task on minimizing the order effect (see the Procedure section for details)].

To investigate the reasons for the possible effects of facial expressions on choice blindness, an exploratory analysis was performed on the “verbal reports.” This analysis was exploratory and was not planned because we did not find any expectation regarding the verbal reports from previous studies (Johansson et al., 2005, 2006). For each participant and each facial expression condition, the verbal reports on the M trials were collected, and the keywords mentioned in these verbal reports were classified into four dimensions. Three dimensions from previous studies were introduced into our study, such as uncertainty, emotionality, and specificity (Johansson et al., 2005, 2006). In addition, considering that Asians were more likely than Westerners to process the faces globally (Miyamoto et al., 2011), personality was employed as the fourth dimension. It referred to instances when participants described faces holistically, such as “friendly,” “self-confident,” and “strict.” Therefore, the verbal reports involved in the present study were categorized into four dimensions, such as uncertainty, emotionality, specificity, and personality. Uncertainty was defined as the frequency of words expressing hesitation and uncertainty in participants' reports. The following words and phrases were considered as the aspect of uncertainty: “probably,” “perhaps,” “I suppose,” and “do not know.” Emotionality was identified as the frequency of words expressing positive and negative emotions. Positive and negative adjectives were included in the aspect of emotionality, such as “happy,” “sad,” and “boring.” Specificity was defined as the frequency of words on facial features reported by the participants, such as “the eyes,” “the eyebrows,” and “the nose.” Personality was defined as the frequency of adjectives that was used to describe faces holistically in participants' reports, for instance, “optimistic,” “easy-going,” and “solemn.” An author and a blind coder carried out all classifications and word-frequency statistics on M trials independently. The inter-coder reliability between the author and the coder was calculated for each of the participant's verbal reports using Holsti's method (Holsti, 1969; Lombard et al., 2002). For Experiment 1, the mean inter-coder reliability on neutral faces was 0.941 (*SE* = 0.016, 95% CI = [0.911, 0.971]) and the mean inter-coder reliability on sad faces was 0.937 (*SE* = 0.017, 95% CI = [0.904, 0.969]). For Experiment 2, the mean inter-coder reliability on neutral faces was 0.930 (*SE* = 0.016, 95% CI = [0.899, 0.961]) and the mean inter-coder reliability on happy faces was 0.958 (*SE* = 0.010, 95% CI = [0.937, 0.978]). In view of the high inter-coder reliability on each facial expression condition for both experiments, the classification results from the author were used for further analysis.

For Experiment 1, to examine the differences in verbal reports between facial expressions on M trials, the word frequency in each of the four dimensions was first compared between neutral and sad facial expressions using the Wilcoxon signed rank test. After finding a significant word-frequency difference on certain dimensions, the word-frequency difference value on this dimension (i.e., neutral-minus-sad difference) and detection rate difference value (i.e., neutral-minus-sad difference) were calculated for each participant. The resulting variables were then subject to a correlation analysis using Spearman rank correlation to explore whether the observed higher choice blindness on sad (as compared to neutral) facial expressions would be reflected by the word-frequency differences in the verbal reports on M trials.

For the purposes of comparison, verbal reports on M trials in Experiment 2 (neutral faces vs. happy faces) were also analyzed using the same methods described above.

## Results

### Experiment 1: larger choice blindness was found for sad faces than neutral faces

#### Detection rate

In Experiment 1, 15 trials of neutral face pairs were presented to 60 participants in one block, and 15 trials of sad face pairs were presented in another block (block order was counterbalanced across participants). Each block consisted of three manipulated trials (M trials) and 12 non-manipulated trials (NM trials). The number of participants who detected 0, 1, 2, and 3 M trials (summation of concurrent detection and retrospective detection) on the neutral facial expression condition and on the sad facial expression condition are summarized in Table 1.

**Table 1:** The number of participants who detected 0, 1, 2, and 3 manipulated trials (summation of concurrent detection and retrospective detection) on neutral and sad facial expression conditions.

	**Detected M trial(s)**			
	**Zero**	**One**	**Two**	**Three**
Neutral faces	27	13	12	8
Sad faces	36	17	6	1

**Figure 1:**
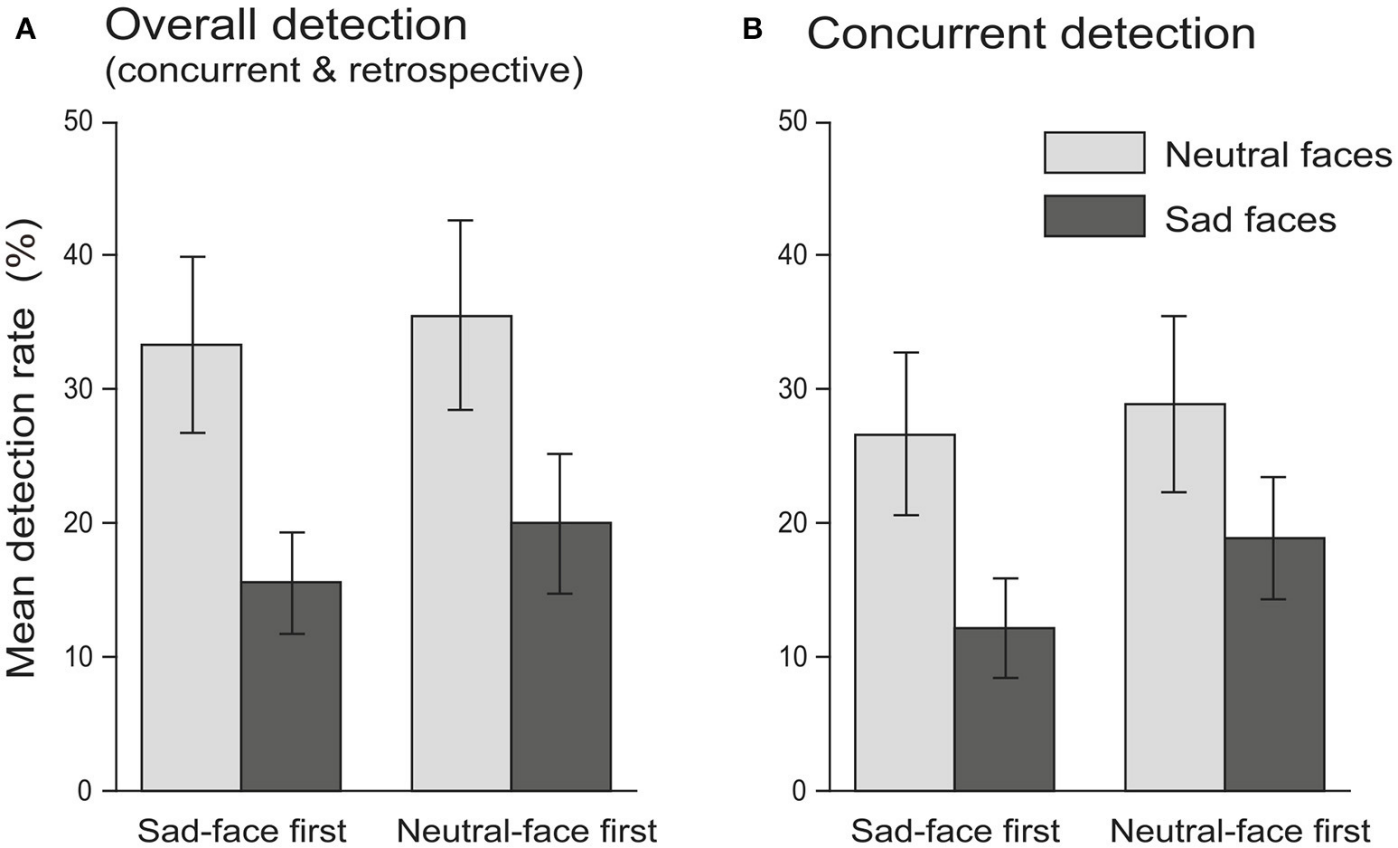
Experiment 1: mean detection rate of M trials as functions of facial expression (within-subject factor: neutral faces vs. sad faces) and block order (between-subject factor: sad-face first group vs. neutral-face first group) for overall detection (**A**, including both concurrent and retrospective detection) and concurrent detection only **(B)**. Note that significant lower detection rate (i.e., larger choice blindness) was found for sad than neutral facial expressions. Error bars in both graphs indicate ± 1 SE.

On an average, the mean detection rate of M trials [the percentage of detected M trials including both concurrent detection and retrospective detection (see the Data analysis section)] on the neutral facial expression condition was 34.44% (*SE* = 4.86%, 95% CI = [24.72%, 44.17%]), which was in agreement with previous observations (e.g., Johansson et al., 2005; Sagana et al., 2014). In contrast, the group mean detection rate on the sad facial expression condition was only 17.78% (*SE* = 3.23%, 95% CI = [11.31%, 24.24%]). The 2 (facial expression: neutral faces vs. sad faces) × 2 (block order: neutral-face first vs. sad-face first) ANOVA for detection rate (see the Data analysis section for details) revealed a highly significant main effect of facial expression [*F*_(1, 58)_ = 11.61, *p* < 0.001, $\eta_{p}^{2}$ = 0.17], with a lower detection rate on sad faces than neutral faces, suggesting larger choice blindness was found for sad facial expressions than for neutral facial expressions. The main effect of block order was not significant [*F*_(1, 58)_ = 0.25, *p* = 0.618, $\eta_{p}^{2}$ = 0.004] (sad-face first group: 24.44 ± 4.70% (mean ± SE), 95% CI = [15.04%, 33.84%]; neutral-face first group: 27.78 ± 4.70%, 95% CI = [18.38%, 37.18%]). The facial expression × block order interaction was not significant [*F*_(1, 58)_ = 0.05, *p* = 0.821, $\eta_{p}^{2}$ = 0.001], indicating that the observed facial expression effect on choice blindness could not be accounted for by block order (Figure 1A).

For the purposes of comparison, the concurrent detection rate (the percentage of immediately detected M trials only) was also subject to the two-way ANOVA mentioned above. As expected, the concurrent detection rate was also found to be significantly lower for sad facial expressions (15.56 ± 3.11% (mean ± SE), 95% CI = [9.33%, 21.78%]) than for neutral (27.78 ± 4.46%, 95% CI = [18.85%, 36.70%]) facial expressions [*F*_(1, 58)_ = 7.84, *p* < 0.01, $\eta_{p}^{2}$ = 0.12]. Once again, neither the main effect of the block order [*F*_(1, 58)_ = 0.49, *p* = 0.486, $\eta_{p}^{2}$ = 0.008] (sad-face first group: 19.44 ± 4.48%, 95% CI = [10.48%, 28.40%]; neutral-face first group: 23.89 ± 4.48%, 95% CI = [14.93%, 32.85%]) nor the facial expression × block order interaction [*F*_(1, 58)_ = 0.26, *p* = 0.613, $\eta_{p}^{2}$ = 0.004] was significant (Figure 1B).

#### Verbal reports

To explore the reasons for the finding that higher choice blindness (i.e., lower detection rate on M trials) was observed on sad facial expressions than neutral facial expressions, an exploratory analysis was performed to explore whether the observed facial expression effect on choice blindness would be reflected by the differences in verbal reports (i.e., the reasons for choice or detection) on M trials. The verbal reports on the M trials were collected, and the keywords mentioned in these verbal reports were classified into four dimensions, namely personality, specificity, emotionality, and uncertainty (see the Data analysis section for details). The word frequency in each of the four dimensions was first compared between neutral and sad facial expressions using the Wilcoxon signed rank test. The results (see Table 2) revealed that, compared to the neutral condition, word frequency mentioned on the emotionality dimension was significantly higher in the sad faces condition, which made sense if sad facial expressions evoke higher emotional arousal than neutral expressions. In contrast, word frequency on specificity was significantly *lower* in the sad condition than the neutral condition, suggesting that participants overall described *less* detailed facial features when seeing faces with sad expressions than when seeing them with neutral expressions.

**Table 2:** Comparisons (*n* = 60) of word frequency on each dimension of verbal reports on M trials between neutral and sad facial expressions.

	**Neutral faces**		**Sad faces**		***Z***	***p***	***d***
	***Mean***	***SD***	***Mean***	***SD***			
Personality	1.02	0.95	0.77	0.87	−1.32	0.185	0.20
Specificity	2.02	1.03	1.48	1.08	−2.59	0.010	0.38
Emotionality	0.12	0.32	0.97	1.10	−4.78	< 0.0001	0.75
Uncertainty	0.58	0.65	0.60	0.69	−0.17	0.865	0.02

*Wilcoxon signed rank test was used to conduct these comparisons*.

The difference values of the word frequency (i.e., neutral-minus-sad difference) on emotionality and specificity dimensions and the difference values of the detection rate (i.e., neutral-minus-sad difference) were then subject to a correlation analysis using the Spearman rank correlation to further explore whether the observed higher choice blindness on sad (as compared to neutral) facial expressions would be related to the word-frequency differences on these dimensions of the verbal reports on M trials. Interestingly, no significant correlation was found between the difference of word frequency on emotionality and the difference of detection rate (*r*_ρ_ = 0.046, *p* = 0.727), but a significant correlation was found between the difference of word frequency on specificity and the difference of detection rate (*r*_ρ_ = 0.335, *p* = 0.009). These results indicated that participants who reported *less* facial features on sad (as compared to neutral) facial expressions also tended to detect *less* M trials (i.e., showed higher choice blindness) on sad (as compared to neutral) facial expressions.

### Experiment 2: moderate choice blindness was found for happy and neutral faces

#### Detection rate

The procedure of Experiment 2 was identical to Experiment 1 with the following exception: 15 trials of neutral face pairs (the same 15 pairs of neutral faces that were used in Experiment 1) and 15 trials of happy face pairs were presented to another 60 participants. The number of participants who detected 0, 1, 2, and 3 manipulated trials (summation of concurrent detection and retrospective detection) on the neutral facial expression condition and that on the happy facial expression condition are summarized in Table 3.

**Table 3:** The number of participants who detected 0, 1, 2, and 3 manipulated trials (summation of concurrent detection and retrospective detection) on neutral and happy facial expressions.

	**Detected M trial(s)**			
	**Zero**	**One**	**Two**	**Three**
Neutral faces	30	13	9	8
Happy faces	32	15	8	5

**Figure 2:**
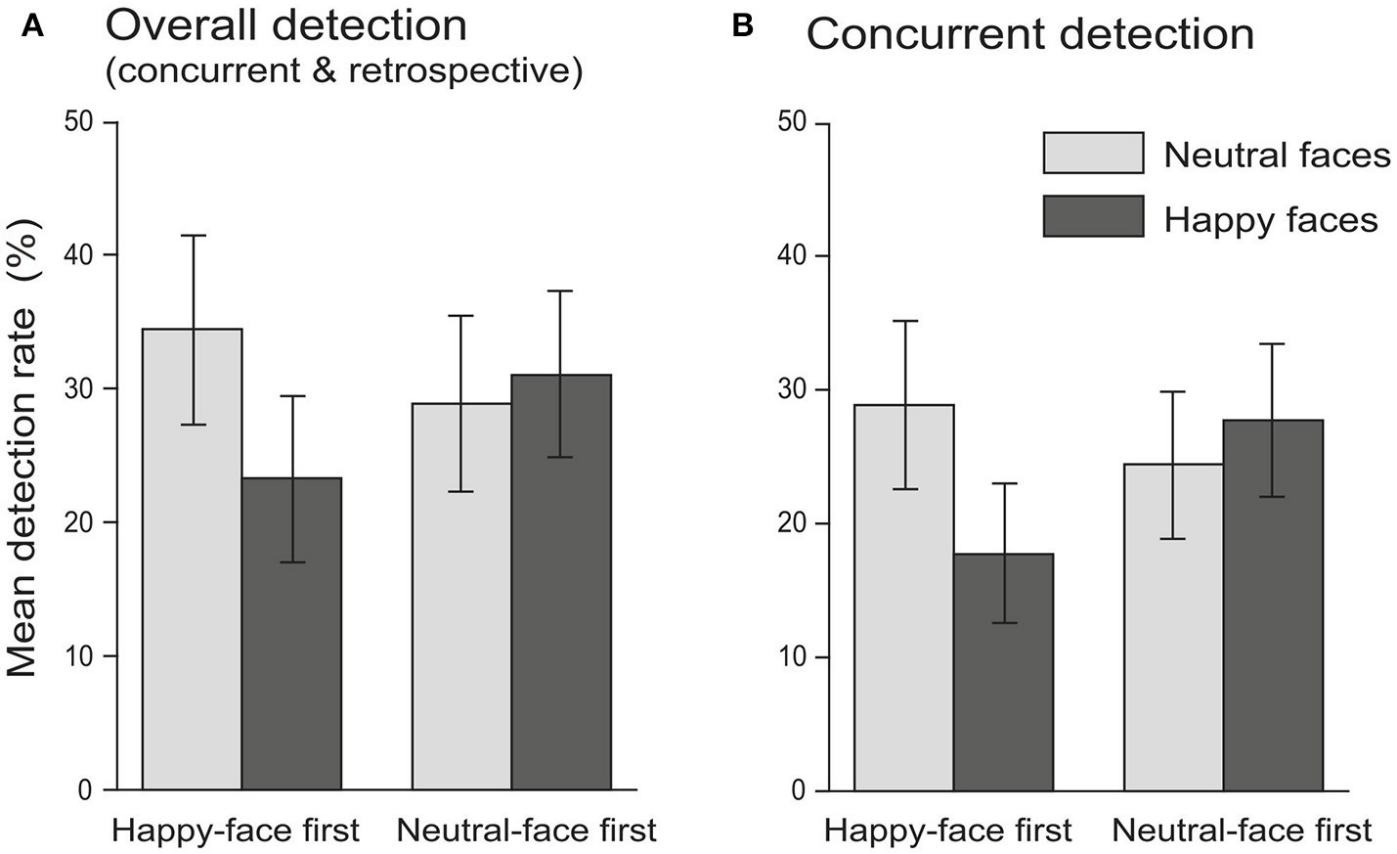
Experiment 2: mean detection rate of M trials as functions of facial expression (neutral faces vs. happy faces) and block order (happy-face first vs. neutral-face first) for overall detection **(A)** and concurrent detection **(B)**. Note that no significant difference in detection rate was found between neutral and happy facial expressions. Error bars in both graphs indicate ± 1 SE.

On an average, the mean detection rate of M trials (including both concurrent detection and retrospective detection) on the neutral facial expression condition in Experiment 2 was 31.67% (*SE* = 4.81%, 95% CI = [22.04%, 41.29%]), which was consistent with the observation in Experiment 1. The group mean detection rate on the happy facial expression condition was 27.22% (*SE* = 4.38%, 95% CI = [18.45%, 35.99%]), which appeared to be comparable to that of the neutral condition. Indeed, the 2 (facial expression: neutral faces vs. happy faces) × 2 (block order: neutral-face first vs. happy-face first) ANOVA for detection rate showed a non-significant main effect of facial expression [*F*_(1, 58)_ = 0.77, *p* = 0.384, $\eta_{p}^{2}$ = 0.01], suggesting that the magnitude of choice blindness on happy facial expressions and on neutral facial expressions did not differ significantly. The main effect of block order was not significant [*F*_(1, 58)_ = 0.02, *p* = 0.885, $\eta_{p}^{2}$ = 0.0004] (happy-face first group: 28.89 ± 5.43% (mean ± SE), 95% CI = [18.02%, 39.76%]; neutral-face first group: 30.00 ± 5.43%, 95% CI = [19.13%, 40.87%]). The facial expression × block order interaction was not significant either [*F*_(1, 58)_ = 1.73, *p* = 0.194, $\eta_{p}^{2}$ = 0.03] (Figure 2A).

Similar to Experiment 1, the concurrent detection rate (the percentage of immediately detected M trials) in Experiment 2 was also subject to the two-way ANOVA mentioned above. The results showed that the main effect of facial expression was also non-significant [*F*_(1, 58)_ = 0.77, *p* = 0.383, $\eta_{p}^{2}$ = 0.01] (neutral faces: 26.67 ± 4.20% (mean ± SE), 95% CI = [18.25%, 35.08%]; happy faces: 22.78 ± 3.90%, 95% CI = [14.97%, 30.58%]). Neither the main effect of block order [*F*_(1, 58)_ = 0.17, *p* = 0.684, $\eta_{p}^{2}$ = 0.003] (happy-face first group: 23.33 ± 4.80%, 95% CI = [13.72%, 32.95%]; neutral-face first group: 26.11 ± 4.81%, 95% CI = [16.49%, 35.73%]) nor the facial expression × block order interaction [*F*_(1, 58)_ = 2.66, *p* = 0.108, $\eta_{p}^{2}$ = 0.04] was significant (Figure 2B).

#### Verbal reports

Although the main finding in Experiment 2 was that the magnitude of choice blindness did not differ significantly between neutral and happy facial expressions, for purposes of comparison, verbal reports on the M trials in Experiment 2 were also analyzed with the same methods as used for Experiment 1 (see above). As shown in Table 4, a significant difference in word frequency between neutral and happy facial expressions was only found in the emotionality dimension, with more frequent emotion-related words reported in the happy than the neutral condition. This provided further evidence for the validity of the face stimuli presented in the present study. The correlation analysis revealed no significant correlation between the difference of word frequency (i.e., neutral-minus-happy difference) on emotionality and the difference of detection rate (i.e., neutral-minus-happy difference) (*r*_ρ_ = 0.001, *p* = 0.994), which was analogous to the pattern observed in Experiment 1. In contrast with Experiment 1, the correlation between word-frequency difference on specificity and detection rate difference was also not significant (*r*_ρ_ = 0.166, *p* = 0.206).

**Table 4:** Comparison (*n* = 60) of word frequency in each dimension of verbal reports on M trials between neutral and happy facial expressions.

	**Neutral faces**		**Happy faces**		***Z***	***p***	***d***
	**Mean**	***SD***	**Mean**	***SD***			
Personality	1.10	0.97	1.25	0.97	−0.69	0.493	0.12
Specificity	1.73	1.09	1.57	1.10	−0.92	0.356	0.12
Emotionality	0.20	0.44	0.53	0.68	−2.85	0.005	0.44
Uncertainty	0.37	0.52	0.24	0.45	−1.72	0.085	0.19

*Wilcoxon signed rank test was used to conduct these comparisons*.

### Follow-up analysis: sad faces were less attractive than happy and neutral faces

Since previous studies have shown that individuals appear less physically attractive when their facial expression is sad compared with when they are neutral or happy (Byrne and Clore, 1970; Mueser et al., 1984; Gelstein et al., 2011; Morrison et al., 2013), we tested whether faces with sad expressions used in our experiments were also perceived as less attractive than happy and neutral expressions. Face stimuli were rated by another 30 subjects for facial attractiveness on a nine-point scale (1 = very unattractive; 9 = very attractive). The faces with happy expressions had a mean attractiveness of 4.62 (*SD* = 1.20; 95% CI = [4.17, 5.07]), the faces with neutral expressions had a mean attractiveness of 4.25 (*SD* = 1.05; 95% CI = [3.86, 4.64]), and the faces with sad expressions had a mean attractiveness of 2.25 (*SD* = 1.10; 95% CI = [1.84, 2.66]). The evaluation of facial attractiveness was subject to a one-way repeated measure ANOVA with facial expressions as the repeated measure with three levels. The result showed that the main effect of facial expression was highly significant [*F*_(2, 58)_ = 73.52, *p* < 0.0001, $\eta_{p}^{2}$ = 0.717]. The *post-hoc* analysis, using the Bonferroni procedure, revealed that both happy and neutral facial expressions were judged as substantially more attractive than sad faces (both *p*s < 0.0001), whereas there was no significant difference in attractiveness between happy and neutral faces (*p* = 0.070).

Most importantly, the three target pairs of happy faces (i.e., M trials on happy expression condition) had a mean attractiveness of 4.57 (*SD* = 1.44; 95% CI = [4.03, 5.10]), the three target pairs of neutral faces had a mean attractiveness of 4.60 (*SD* = 1.07; 95% CI = [4.20, 5.00]), and the three target pairs of sad faces had a mean attractiveness of 2.18 (*SD* = 1.16; 95% CI = [1.74, 2.61]). A similar ANOVA for the evaluation of facial attractiveness on M trials also revealed a highly significant main effect of facial expression [*F*_(2, 58)_ = 59.21, *p* < 0.0001, $\eta_{p}^{2}$ = 0.671]. The *post-hoc* analysis again showed that both the target pairs of happy and neutral faces were perceived as more attractive than the sad faces (both *p*s < 0.0001), and no significant difference was observed between target pairs of happy and neutral faces (*p* = 0.988). These results indicate that sad faces were perceived as less attractive than happy and neutral faces in the current study.

## Discussion

Previous choice blindness studies have found a couple of factors that influenced the magnitude of choice blindness, such as similarity (Sagana et al., 2013), preference strength (Somerville and McGowan, 2016), and presentation time (Johansson et al., 2005). Notably, only faces with neutral facial expressions were used as stimuli to explore contributory factors in the abovementioned studies, and previous studies, to our knowledge, have not yet investigated the potential effect of facial expressions on choice blindness. Here, we explored whether the CB effect would also be affected by facial expressions. Using sad, neutral, and happy faces, the current study investigated this issue in a typical choice blindness paradigm. The results revealed that, on average, nearly a third of M trials were detected on neutral facial expression, which was in line with previous studies (Johansson et al., 2005; Sagana et al., 2014). More importantly, a lower detection rate of M trials (i.e., larger CB) was observed on sad expressions than on neutral expressions (Experiment 1), whereas the mean detection rate did not differ significantly between happy expressions and neutral expressions (Experiment 2). These results indicated that choice blindness can be affected by facial expression, but the facial expression effect occurred only for sad (but not happy) expressions.

The participants' task in the current study was to choose which face in each pair was more attractive and then report the reasons for their choices. In other words, the choice that participants made on each trial was probably based on their evaluation of facial attractiveness. On one hand, many previous studies have shown that individuals appear less physically attractive when their facial expression is sad compared with neutral or happy expressions (Byrne and Clore, 1970; Mueser et al., 1984; Gelstein et al., 2011; Morrison et al., 2013). Happy and neutral facial expressions were perceived to be more attractive expressions probably because these “safe” expressions (i.e., pleasure and neutral) were more likely to signal a desire to approach, cooperate, and interact socially with participants (Rhodes, 2006; Morrison et al., 2013). In contrast, sad facial expressions were more likely to be a distressing cue, which resulted in participants keeping their distance away from the sad expression and reacting with avoidance-related behaviors (Seidel et al., 2010). On the other hand, it has also been found that more attractive faces (i.e., happy or neutral faces) are recognized more easily in a recognition task (Marzi and Viggiano, 2010). This attractiveness effect on recognition was found to be related to enhanced reward signals processed by the orbitofrontal cortex, which contributed to both the encoding and retrieval processing stages (Tsukiura and Cabeza, 2008; Marzi and Viggiano, 2010).

Taking these previous findings together, it is reasonable to infer that both happy and neutral faces presented in the current study might be subjectively more attractive (as compared to sad faces) for participants, which, in turn, might make them relatively more willing to process the facial features on both happy and neutral faces. Therefore, an intermediate magnitude of choice blindness was found for both happy and neutral expressions, and no significant difference in choice blindness was observed between them. In contrast, sad faces in the present study might be perceived as a less attractive facial expression, which might make participants relatively less willing to further process and encode the facial feature information on sad faces. Hence, a substantially larger choice blindness was observed for sad expressions.

The above-mentioned inference, on the one hand, was further supported by our follow-up analysis of facial attractiveness, as happy and neutral expressions were indeed perceived as more attractive expressions than sad faces in the current study (see the Results section for details). On the other hand, this inference was also supported by the exploratory analysis of the verbal reports on the M trials. Specifically, in Experiment 1, the word frequency reported on the specificity dimension was found to be significantly lower for sad faces than neutral faces, and a significant correlation was found between the differences in word frequency (i.e., neutral-minus-sad difference) on specificity and the differences in detection rate (neutral-minus-sad difference). That is, participants who reported *fewer* facial features on sad (as compared to neutral) faces also tended to detect *fewer* M trials (i.e., show larger choice blindness) on sad (as compared to neutral) facial expression. These results of the verbal reports in Experiment 1, along with the results of the attractiveness analysis that sad expressions were indeed perceived as less attractive, are consistent with the above proposal that the less attractive sad expression might inhibit participants from further processing and encoding the detailed facial features on sad faces and thereby lead to a substantially larger choice blindness.

In addition, an analysis of the verbal reports in Experiment 2 revealed that no significant difference was found in word frequency on the specificity dimension between happy and neutral faces, and no significant correlation was observed between the difference in word frequency (i.e., neutral-minus-happy) on specificity and the difference in detection rate (neutral-minus-happy). These results of the verbal reports in Experiment 2, along with the results of the attractiveness analysis that there was no significant difference in attractiveness between happy and neutral faces, indicated that there was no significant difference in choice blindness between happy and neutral faces, which appeared to result from happy faces being perceived as attractive as neutral faces, which leads to detailed facial features on happy faces being processed as comparably as that on neutral faces. It should be noted that although more processing of detailed facial features may not be necessarily synonymous with better recognition memory, encoding detailed facial features would become particularly important when detecting changes between two faces with the same facial expression and intermediate similarity in the present study.

One possible contamination to our findings would be that the observed facial expression effect on choice blindness might have simply resulted from the difference in the similarity of the paired faces between sad and neutral expressions. Previous studies showed that choice blindness was significantly increased for faces with high similarity (Sagana et al., 2013), and the effect of similarity was also observed on choice blindness for other modalities, such as taste and smell (Hall et al., 2010). However, the physical similarity of paired faces for each facial expression condition was kept constant at an intermediate level (i.e., 4~5 point on a nine-point scale) in the current study. More importantly, no difference in similarity was found between sad and neutral expressions in the current study (see the Stimuli section for details). Therefore, the results found in the current study may not be due to the similarity effect on choice blindness.

Another possible contamination to our findings would be that the lower detection rate of the sad expression than the neutral expression was driven, at least partially, by the order effect, as sad face pairs and neutral face pairs were assigned into two distinct blocks. However, this was also unlikely to be the case because the potential effect of block order had been controlled in the present study by counterbalancing block order across participants and by introducing an interference task between the two blocks to minimize the effect of block order. More importantly, our results of non-significant facial expression × block order interaction on detection rate (see Results section for details) demonstrated that the observed facial expression effect could not be accounted for by block order, and the between-block interference task is valid on attenuating the order effect.

In addition, one might argue that the 4-s presentation of each facial pair used in the present study was not sufficient for participants to process the presented faces and choose one that they found more attractive. However, previous studies have shown that people are remarkably fast at forming opinions about facial appearance (Todorov et al., 2005) and are able to evaluate facial attractiveness after as short of an exposure as 100 ms (Willis and Todorov, 2010). It is also worth mentioning that Johansson et al. (2005) have already shown that even a 2-s presentation time in the choice blindness paradigm is sufficient for a great majority of participants to form a preference for face pairs. Hence, the 4-s presentation time used in the present study is substantially enough for participants to process the presented faces and form an opinion about aesthetic preference.

## Conclusion

In summary, the current study explored whether CB would be affected by facial expressions and found that a lower detection rate of M trials (larger CB) was observed for sad expressions than neutral expressions (Experiment 1), whereas the mean detection rate did not differ significantly between happy expressions and neutral expressions (Experiment 2). The exploratory analysis of the verbal reports showed that participants who reported fewer facial features on sad (as compared to neutral) expressions also tended to detect fewer M trials on sad (as compared to neutral) facial expression conditions. We believe that the present results provide insight for understanding choice blindness, with the major findings being that a higher choice blindness was observed on sad trials, which might be due to the processing inhibition of detailed facial features by the less attractive sad expressions as compared to the neutral expressions.

## Author contributions

YW contributed to experimental design, data collection, data analysis, and paper writing. SZ contributed to data analysis and paper writing. ZZ and WF contributed to experimental design, data analysis, and paper writing.

### Conflict of interest statement

The authors declare that the research was conducted in the absence of any commercial or financial relationships that could be construed as a potential conflict of interest.

## Appendix

### Post experiment interview

- Did you notice the emotions from the photographs?
- Did you feel down or happy by looking at the photographs?
- Did you find anything odd about the task?
- Did you notice anything strange of the photographs?
- We're planning another study using magic—when people pick the face they prefer we're going to use a magic trick to present them with the picture that they didn't pick. Do you think you would notice if we did this?
